# Anionic Exchange Membrane for Photo-Electrolysis Application

**DOI:** 10.3390/polym12122991

**Published:** 2020-12-15

**Authors:** Carmelo Lo Vecchio, Alessandra Carbone, Stefano Trocino, Irene Gatto, Assunta Patti, Vincenzo Baglio, Antonino Salvatore Aricò

**Affiliations:** Institute for Advanced Energy Technologies “Nicola Giordano”—CNR-ITAE, Via Salita S. Lucia sopra Contesse 5, 98126 Messina, Italy; stefano.trocino@itae.cnr.it (S.T.); irene.gatto@itae.cnr.it (I.G.); assunta.patti@itae.cnr.it (A.P.); vincenzo.baglio@itae.cnr.it (V.B.); antonino.arico@itae.cnr.it (A.S.A.)

**Keywords:** anion-exchange membrane, ionic conductivity, ionomer, photo-electro-chemical applications, tandem cell

## Abstract

Tandem photo-electro-chemical cells composed of an assembly of a solid electrolyte membrane and two low-cost photoelectrodes have been developed to generate green solar fuel from water-splitting. In this regard, an anion-exchange polymer–electrolyte membrane, able to separate H_2_ evolved at the photocathode from O_2_ at the photoanode, was investigated in terms of ionic conductivity, corrosion mitigation, and light transmission for a tandem photo-electro-chemical configuration. The designed anionic membranes, based on polysulfone polymer, contained positive fixed functionalities on the side chains of the polymeric network, particularly quaternary ammonium species counterbalanced by hydroxide anions. The membrane was first investigated in alkaline solution, KOH or NaOH at different concentrations, to optimize the ion-exchange process. Exchange in 1M KOH solution provided high conversion of the groups, a high ion-exchange capacity (IEC) value of 1.59 meq/g and a hydroxide conductivity of 25 mS/cm at 60 °C for anionic membrane. Another important characteristic, verified for hydroxide membrane, was its transparency above 600 nm, thus making it a good candidate for tandem cell applications in which the illuminated photoanode absorbs the highest-energy photons (< 600 nm), and photocathode absorbs the lowest-energy photons. Furthermore, hydrogen crossover tests showed a permeation of H_2_ through the membrane of less than 0.1%. Finally, low-cost tandem photo-electro-chemical cells, formed by titanium-doped hematite and ionomer at the photoanode and cupric oxide and ionomer at the photocathode, separated by a solid membrane in OH form, were assembled to optimize the influence of ionomer-loading dispersion. Maximum enthalpy (1.7%), throughput (2.9%), and Gibbs energy efficiencies (1.3%) were reached by using n-propanol/ethanol (1:1 wt.) as solvent for ionomer dispersion and with a 25 µL cm^−2^ ionomer loading for both the photoanode and the photocathode.

## 1. Introduction

According to the European Strategy Energy Technology (EU SET) plan, by 2050 at least 65% of electric energy should derive from renewable energy sources and, furthermore, CO_2_ emissions related to the energy production should be reduced by 50%. From this perspective, a drastic reduction in the dependence from fossil fuels could be realized by exploiting all abundant renewable sources that nature reserves: sun [1,2,3], wind [4,5], water [6,7], and geothermic [8,9]. These technologies have recently experienced large-scale commercialization, and generate electric energy by directly converting the energy of renewable sources. However, the main drawbacks are related to their relative intermittence, causing issues in terms of balancing grid processes.

Energy produced by the sun in one hour is equal to the amount necessary for the world’s human population in one year [10]. Thus, storing energy through the production of solar fuel (hydrogen by water-splitting or hydrocarbons by carbon dioxide reduction) and generating energy from them, when it is necessary, has become a “green” challenge [11,12,13,14].

Photo-electro-chemical (PEC) water-splitting (WS) is a process in which oxygen and hydrogen evolve at photoanode and photocathode, respectively, using a liquid electrolyte, generally based on concentrated hydroxides [15,16,17,18]. Efficiency and durability to produce pure H_2_ by photo-electrolysis may be improved by employing a solid polymer-electrolyte between the electrodes as a gas separator. This separation for polymer-electrolyte membrane fuel cells (PEMFCs) and their subcategories [19,20,21,22] has been known for many years whereas the employment of a solid-membrane electrolyte has been less studied for PEC applications [23,24]. Its implementation in a cost-effective tandem PEC architecture, able to capture a significant portion of the solar irradiation, is structured as photoanode/membrane/photocathode and the working mechanism has been discussed in detail in some recent work [25,26].

**Figure 1:**
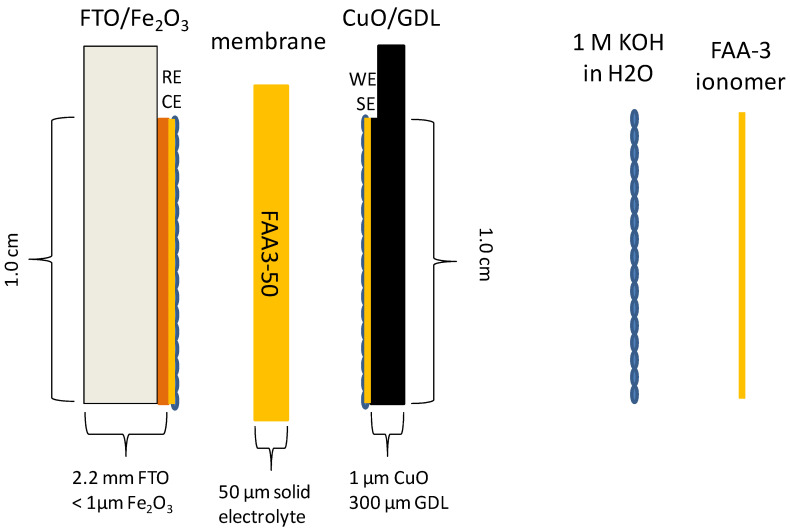
Sketch of the photo-electro-chemical cell for water-splitting.

As shown in Figure 1, photoelectrodes are based on earth-abundant metal oxides such as α-Fe_2_O_3_ (hematite) at the photoanode (PA), supported on fluorine tin oxide (FTO) substrate and CuO at the photocathode (PC) deposited on a gas diffusion layer (GDL) [27]. The polymer-electrolyte approach requires an effective interfacial contact between polymer-electrolyte and photoelectrodes able to permit an efficient ionic percolation.

Infiltration of ion clusters, obtained by dispersing the ionomer shredded film in alcoholic solution, into the nanostructured semiconductor’s layer forms an extended interface with the metal oxides. This is likely facilitated by the nanocolumnar or nanofibrous nature of the oxide material, which should allow a more straightforward penetration of the ion clusters throughout the entire thickness of the nanostructured electrode. In the sketch, connections for PEC measurements are represented with RE (reference electrode) and CE (counter electrode) at the photoanode and with WE (working electrode) and SE (sensing electrode) at the photocathode.

In summary, the planned research targets for the polymer-electrolyte membrane/ionomer structure are the following:

- Suitable ionic conductivity in the range of 10–50 mS cm^−1^.
- Low gas crossover. Hydrogen/oxygen permeation smaller than 10^−8^ mol cm^−2^ min^−1^.
- High water permeation to allow for proper water management between cathode and anode according to the main conduction mechanism.
- Availability of ionomer dispersions to allow for an extension of the ionomer/oxide interface.
- Robust mechanical and thermal properties. Capability to sustain high temperatures (up to 85 °C).
- Optical transparency in the useful range of wavelengths.
- Substantial photo-response of the semiconductor electrodes in contact with the polymer material.
- Semiconductor corrosion mitigation properties.
- Low cost and wide availability of the base components.

Pros and cons of using protonic or anionic membranes for separating the electrodes, as well as the issues related to their transparency to use them in a tandem photo-electrolysis cell, have been evaluated [24]. A significant increase either in photocurrent or spontaneous cell photovoltage for the anionic-membrane-based cell was observed and correlated with better reaction kinetics. Thus, modified anionic Fumasep membrane (FAA3-50 from Fumatech) was identified instead of conventional protonic Nafion membrane.

The anionic electrolyte (FAA3-50) is essentially a quaternary ammonium-based side-chain-type membrane modified with nitrogen functionalities/bringing groups to provide an extended network of hydrogen bonds for enhanced hydroxide ion transport. Measurements of the resulting ionic conductivity and hydrogen–oxygen crossover were carried out and membranes were also assessed in terms of light transmission for the tandem semiconductor cell concept. Furthermore, ionomer dispersions (FAA3-ION), obtained by dissolving the solid membrane in appropriate solvents, were also employed to increase the electrode-electrolyte interface and favor the interaction of the electrode with the solid polymer membrane. Their suitable deposition procedure along with nanostructured electrode surface was properly improved in terms of ionomer loading and use of solvents in the dispersion, resulting in a layer able to increase the stability of hematite and CuO-based electrodes. The anionic polymer membrane and ionomer materials are designed to have a high mechanical integrity and low gas crossover, to operate under high internal pH and in a highly conductive environment for OH^-^ ions.

The novelty of this work is related to a detailed study of the polymer-electrolyte membrane to evaluate its suitability for PEC applications. In particular, IEC, optical transparence, anion conductivity, and hydrogen crossover through the membrane were investigated. Furthermore, different ionomer loading (10–45 μL cm^−2^) and dispersions either in alcoholic or hydroalcoholic solvents were optimized in a complete tandem PEC cell.

## 2. Materials and Methods

### 2.1. Membrane

Fumasep membrane (FAA3-50 from Fumatech, Bietigheim-Bissingen, Germany), characterized by a thickness of 50 μm and based on a brominated polysulfone backbone with quaternary ammonium side chain groups, was selected and characterized for this application.

Commercial FAA3-50, received in bromide form, was exchanged in 1 M NaCl solution for 72 h. Before electro-chemical tests of tandem PECs, the anionic membrane, in chloride form, was further conditioned in a fresh 1 M KOH solution for 24 h.

### 2.2. Ionomer Preparation

The ionomer dispersion was achieved by solubilizing the ionomer in a solid form (FAA3-shredded film) in a mixture of solvents [28]. An alcoholic solution of n-propanol and ethanol (1:1 wt.) was prepared and the FAA3 ionomer was solubilized at room temperature under stirring to have ~5 wt.% dispersion. The concentration was checked by the ratio of the dry mass of a specific amount of desiccated polymer solution over the wet mass.

### 2.3. Determination of Ion-Exchange Capacity (IEC)

To calculate the total amount of ions contained in the membrane, a back-titration based on the Volhard method was carried out [29]. This method was able to recognize the halides coordinated to the quaternary ammonium groups, in particular Br^−^ and Cl^−^. It was performed on both samples, i.e., the FAA3-50 as-received (Br^−^ form) and after the exchange in 1 M NaCl solution. The detailed procedure is described in the following steps:

(1) drying the membrane in an oven at 50 °C for 2 h under vacuum condition (1000 mbar);
(2) immersing the dried sample in 0.1 M NaNO_3_ for 48 h at room temperature, afterwards removing the membrane from the solution;
(3) adding 5 mL of 0.1 M AgNO_3_ and 5 drops of Fe(NO_3_)_3_ (11 wt.%), used as an indicator;
(4) back-titration with 0.1 M KSCN until the equivalent point.

The IEC was calculated as follows in Equation (1):

IEC = (V_AgNO3_ − V_KSCN_) [KSCN]/m_dry_ (1)

Moreover, to calculate the number of halides converted into OH^−^ ions, responsible for the photo-electrolysis process, an acid–base back-titration is necessary. The detailed procedure is described in the following steps:

(1) immersing the dried sample in alkaline solution at room temperature for a specific time, after removing the membrane from the solution and washing to remove the excess of hydroxide ions;
(2) drying the membrane in oven at 50 °C for 2 h under vacuum (1000 mbar);
(3) immersing the membrane in HCl 0.01 M for 24 h at room T, then removing the membrane from the solution;
(4) back-titration with NaOH 0.01 M.

The IEC was calculated as follows in Equation (2):

IEC = (V_HCl_ − V_NaOH_) · [NaOH]/m_dry_ (2)

### 2.4. UV-Vis-NIR Detection

UV-Visible-NIR measurements were carried out with Cary Win UV 6000 (Agilent Technologies, Santa Clara, CA, USA) in the spectral range 2000–200 nm. Chloride (FAA3-50 Cl^−^), bromide (FAA3-50 Br^−^) and hydroxide (FAA3-50 OH^−^)-based membranes were tested after zeroing the dual-beam instrument in air.

### 2.5. In-Plane Anion Conductivity Measurements

The in-plane anion conductivity of samples with different counter-ions (Br^−^, Cl^−^, OH^−^) was carried out with 4 electrodes by electro-chemical impedance spectroscopy (EIS) method. The EIS parameters for the measurement were: 100 kHz-1 Hz of frequency range and 50 mV of amplitude. The measurements were carried out in the range of temperature 30–60 °C, flowing humidified N_2_ (100% relative humidity, RH) at room pressure.

### 2.6. Electro-Chemical Characterizations

#### 2.6.1. Assessment of Hydrogen Crossover

The H_2_ crossover of the membranes was measured in 25 cm^2^ fuel cell/electrolyzer configuration [30,31] by a linear sweep method, feeding hydrogen to the anode, operating both as counter and reference electrode, and nitrogen to the cathode (working electrode). The used operative conditions are the following: 40–60 °C temperature range; 100% relative humidity (RH); 1 bar abs pressure, 100 mL/min and 260 mL/min flow rate for N_2_ and H_2_, respectively. The cell potential was varied from 0 to 0.8 V with a scan rate of 4 mV/s. The current value at 0.4 V was used for crossover calculation. Home-made electrodes were prepared by spraying the catalytic ink onto a commercial GDL Sigracet (SGL Carbon, Drachenburgstraße Bonn, Germany). The catalytic ink was obtained by mixing the 40% Pt/C (Alfa Aesar, Erlenbachweg Kandel, Germany) with a 20 wt.% of dry FAA3 ionomer (5 wt.% alcoholic solution). A Pt loading of 0.5 mg/cm^2^ for both the anode and the cathode side was used.

#### 2.6.2. Photo-Electro-Chemical Test

The PEC components were the following: (i) Photoanode based on titanium-doped hematite deposited over FTO glass; (ii) an anionic membrane FAA-3 in hydroxide form as the electrolyte [25,26]; (iii) photocathode based on copper oxide deposited on a Sigracet 35BC (SGL Carbon) substrate (previously hydrophobized with fluorinated ethylene propylene, FEP, at a loading of 7 wt.%) [27]; (iv) the ionomer dispersion was deposited over the photoelectrodes and after drying a conditioning step in 1 M KOH for 1 h was carried out.

The tandem PEC cell (1 cm^2^) was assembled taking into account the best results obtained presently by our group as published in a recent paper [27]. The anionic membrane in hydroxide form (FAA3-50) was assembled between photoanode (Ti-doped hematite + ionomer) and photocathode (CuO/GDL + ionomer), as depicted in Figure 1 and described in previous papers [25,26]. The sandwiched cell was clamped at the sides providing a 0.25 cm^2^ active area. The light source in the PEC measurement was 1 sun illumination. The cell was placed in horizontal mode and illuminated from the top with the light coming directly onto the photoanode. The tandem concept was fulfilled because the highest wavelength range of the total irradiation is not absorbed either by wide energy gap hematite photoanode (2.1 eV) or by the transparent membrane and it can thus reach the low energy gap photocathode.

On–off polarization tests were carried out by sweeping the potential between the open circuit potential (OCP) value up to a bias of −1.3 V and interchanging 5 s under illumination to 5 s dark. Reference (RE) and counter (CE) electrodes were connected to the FTO at photoanode whereas working (WE) and sensing (SE) electrodes were linked to the GDL at the photocathode. Thus, the sign of the recorded photocurrent and the potential bias are reported as negative (reverse current and applied bias) as in the case of a photodiode. The photocurrent measured between the OCP and the short circuit (i.e., 0 V) is driven by the illumination only (spontaneous photocurrent), and in this region the potential is positive (spontaneous photovoltage). On the other hand, in the negative potential region, an external bias-assisted photocurrent occurs.

The solar to hydrogen efficiency is calculated as follows in Equations (3)–(5):

Enthalpy efficiency η_Enth_ = I_p_ (ΔH/nF − E_bias_)/P_in_ = I_p_ (E_tn_ − E_bias_)/P_in_ (3)

Gibbs Energy efficiency η = I_p_ (ΔG/nF − E_bias_)/P_in_ = I_p_ (E_rev_ − E_bias_)/P_in_ (4)

Throughput efficiency η_throughput_ = I_p_ (ΔH/nF) /(P_in_ + I_p_ E_bias_) = I_p_ E_tn_/(P_in_ + I_p_ E_bias_) (5)

where E_tn_ = 1.48 V, E_rev_ = 1.23 V, E_bias_ ≡ V, P_in_ ≡ mW cm^−2^ and I_p_ = I_light_ − I _dark_ ≡ mA cm^−2^.

## 3. Results and Discussion

### 3.1. Membrane Characterization

**Figure 2:**
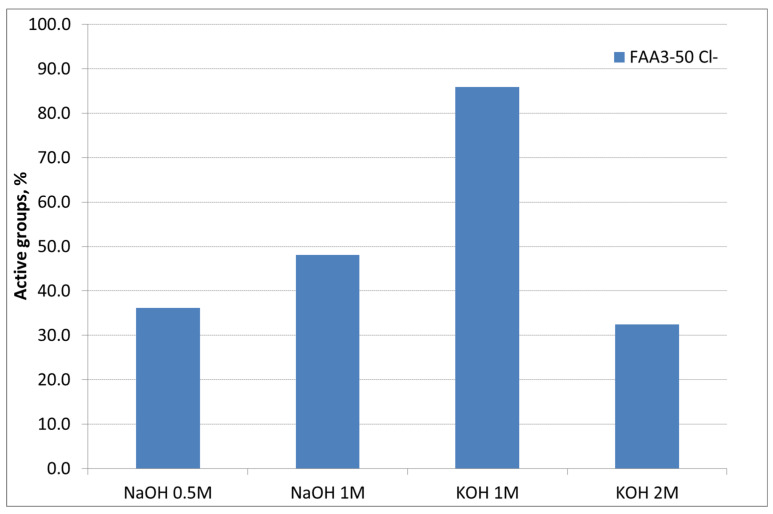
Influence of base type and concentration during the ion-exchange process.

A preliminary study about the influence of alkaline solution (KOH or NaOH), and its concentration during the membrane ion-exchange process, for samples exchanged in 1 M NaCl solution for 72 h (Cl^−^ form) was performed (Figure 2). The first study was carried out using NaOH as an alkaline medium for the ion exchange. It is evident that increasing the molarity and maintaining the time as a constant at 24 h a higher conversion of Cl^−^ groups can be achieved. In addition, maintaining the concentration at 1 M, the exchange with KOH instead of NaOH provided a higher conversion of the groups and the highest IEC value of 1.59 meq/g was reached against the nominal value of 1.85 meq/g. A further increase of concentration up to 2 M produced a drop in the conversion due to a degradation of the functional groups in strong alkaline media [32]. Accordingly, 1 M KOH was selected as the best alkaline solution concentration for ion exchange.

Different titrations were carried out on as-received (Br^−^ form), chloride exchanged in NaCl (Cl^−^ form), and hydroxide exchanged in KOH (OH^−^ form) samples, to define the need to convert the bromide ions into chloride before having the hydroxide form, useful for the final application, instead of a direct exchange from Br^−^ to OH^−^. In Table 1 the comparison of the IECs and the corresponding active groups as a function of the counter-ion is reported. The active groups are calculated as the difference between the nominal IEC (1.85 meq/g) and those calculated by Volhard and acid–base back-titration.

Given an excess of bromide ions results available for the ion exchange (Volhard method), this behavior suggests some residual bromide ions during the preparation process of the membrane that are not really involved in the ion-exchange process. Thus, the same characterizations were carried out on the sample exchanged in NaCl, to verify if this treatment can also purify the membrane from production of residuals (second raw of Table 1).

In this case, the nominal value was reached and 100% of active groups are available for the ion exchange. The exchange in alkaline media produced a reduction of IEC down to 1.59 meq/g and the corresponding active groups reached only 85% probably due to a degradation process in alkaline media. From such data, it is possible to conclude that the membrane must be exchanged in NaCl before being used to have the dual effect of anion exchange and purification.

**Figure 3:**
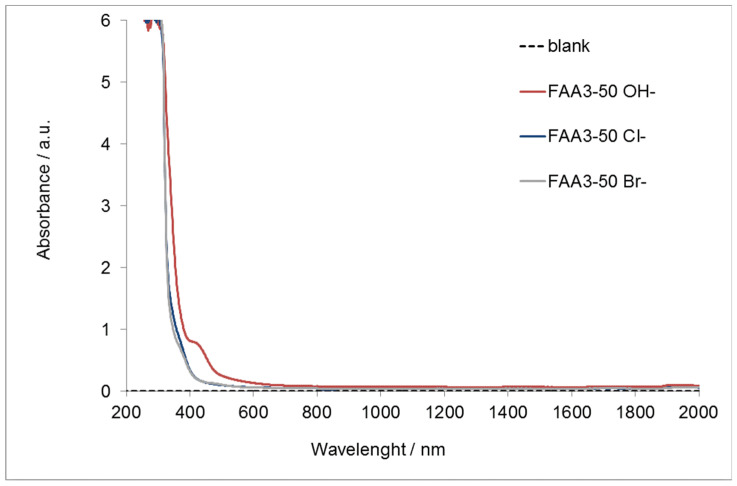
UV-Vis–NIR absorption for FAA3-50 in chloride, bromide, or hydroxide form.

Figure 3 reports the UV-vis spectrum of the membrane with the different counter-ions.

It is evident that the chloride and bromide membranes are almost completely transparent to visible light and a strong absorption appears only in the ultra-violet region (200–400 nm). On the other hand, FAA3-50 OH^−^ is transparent to the light over the wavelength of 600 nm. However, the photoanode adsorbs the light in the orange visible region and for this reason the alkaline membrane appears to be transparent to the diffused light from the photoanode. This makes the FAA3-50 membrane a good candidate for the photo-electrolysis application.

**Figure 4:**
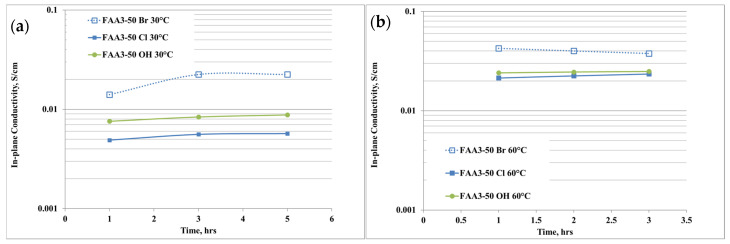
(**a**,**b**) Anion conductivity of the membrane with different counter-ions for the quaternary ammonium at (**a**) 30 °C, (**b**) 60 °C.

The anion conductivity of the membrane with different counter-ions for the quaternary ammonium is shown in Figure 4a,b. “Br” indicates the as-received membrane, “Cl” the membrane exchanged in NaCl solution while “OH” indicates the hydroxide form after the exchange in KOH solution.

The ion conductivity was measured at 30 °C and 60 °C, i.e., the temperature range of interest for this application, and, according to the physico-chemical data, at 30 °C the highest conductivity was achieved for the Br^−^ sample (Figure 4a). This effect is due to the presence of an excess of Br^-^ ions that cannot be distinguished during the measurements. The reduction of conductivity after the exchange in NaCl solution is due to the purification process and it is in accordance with the IEC results. After the conversion in hydroxide, the anion conductivity further increased, according to a higher mobility of OH^−^ ions than chloride ones. It is also noticeable that the conductivity slightly increases with the time, so a conditioning period is necessary to reach an equilibrium phase. Increasing the temperature to 60 °C (Figure 4b) produces an increase of the conductivity and the trend remained the same as 30 °C, with a maximum of about 25 mS/cm after 3 h of equilibration time for FAA3-50 OH.

Characterization in a 25 cm^2^ single cell was carried out to quantify the H_2_ crossover, as reported in Table 2. In Figure S1 the LSV plots at the 3 different temperatures are reported. Considering that the cell was fed with 0.173 mL/s cm^2^ H_2_ flow during the measurement, the crossover percentage (last column of Table 2) can be determined to evaluate the amount of H_2_ permeated through the membrane.

The crossover was in the order of magnitude of 10^−1^ mA/cm^2^, one order of magnitude lower than the U.S. Department of Energy (DoE) target suggested for fuel cell (2 mA/cm^2^) [33]. In addition, less than 0.1% of H_2_ fed to the cell is permeated through the membrane. Considering that the hydrogen produced during the photo-electrolysis process is quite low, the crossover value can be considered negligible.

### 3.2. Photo-Electro-Chemical Test for Membrane Assessment in Full-Cell Configuration

**Figure 5:**
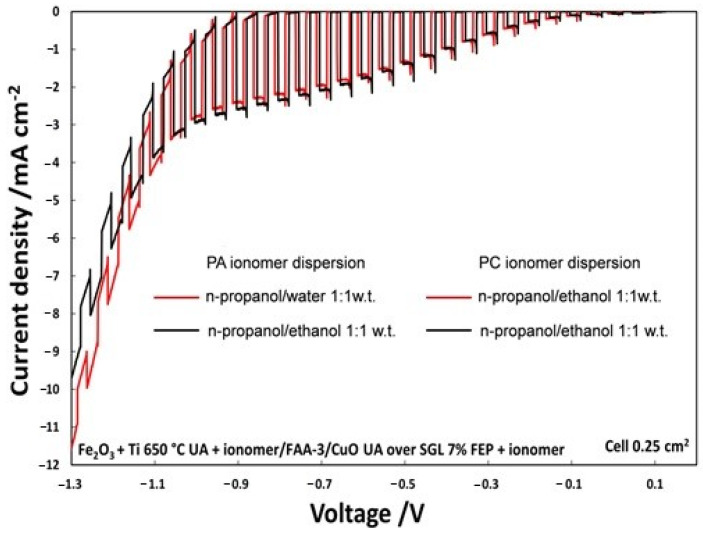
Polarization chopped curves for the two different ionomer dispersion at photoanode.

The on–off illumination experiments, shown in Figure 5, from OCP to an external bias of −1.3 V, compares two PECs in which only the solvent of photoanode ionomer dispersion, hydroalcoholic n-propanol/water (1:1 wt.) or alcoholic n-propanol/ethanol, was varied. The photocurrent density, with an applied voltage of −0.6 V, was 1.9 mA/cm^2^ using n-propanol/ethanol (1:1 wt.); however, it was 1.8 mA/cm^2^ for the n-propanol/H_2_O (1:1 wt.) mixture. Even if photocurrent density is not too much affected by the solvent, the alcoholic solution was selected for the successive studies for practical purposes.

**Figure 6:**
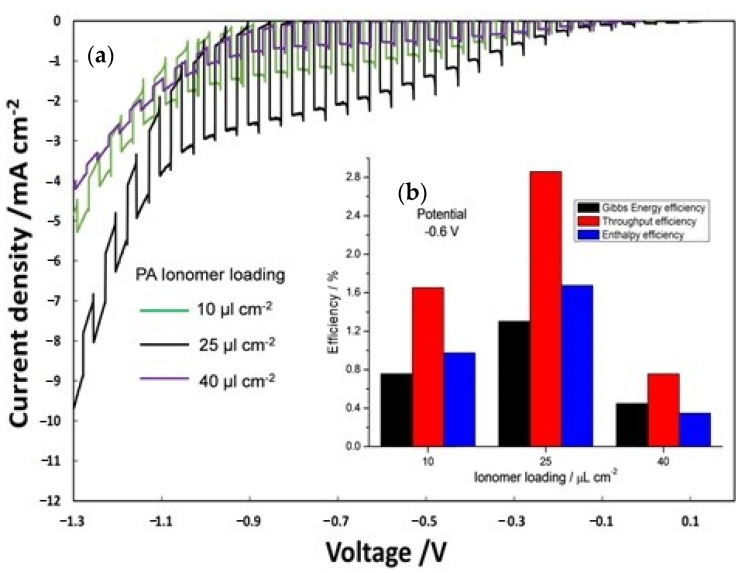
(**a**) On–off polarization curves by varying ionomer loading at photoanode; (**b**) efficiency versus photoanode ionomer loading.

Furthermore, the influence of ionomer content was evaluated (Figure 6) by analyzing different ionomer loading (10, 25 and 40 µL cm^−2^) on the photoanode (PA) and keeping constant at 25 µL cm^-2^ the photocathode ionomer content. The best results in terms of photocurrent density (Figure 6a) were obtained with an ionomer loading of 25 µL cm^−2^. Figure 6b displays the efficiency of the PEC cells, calculated at −0.6 V by equation 3, 4, and 5 as a function of different ionomer loadings on PA. Efficiency of the three different PECs is clearly dependent on a proper amount of ionomer (25 µL cm^-2^) reaching a maximum of enthalpy (1.7%), throughput (2.9%) and Gibbs energy efficiencies (1.3%). A lower amount of ionomer (10 µL cm^−2^) is not sufficient to get an extended interface with Ti-doped hematite surface, whereas a larger deposition (40 µL cm^−2^) causes a loss of electric conductivity.

**Figure 7:**
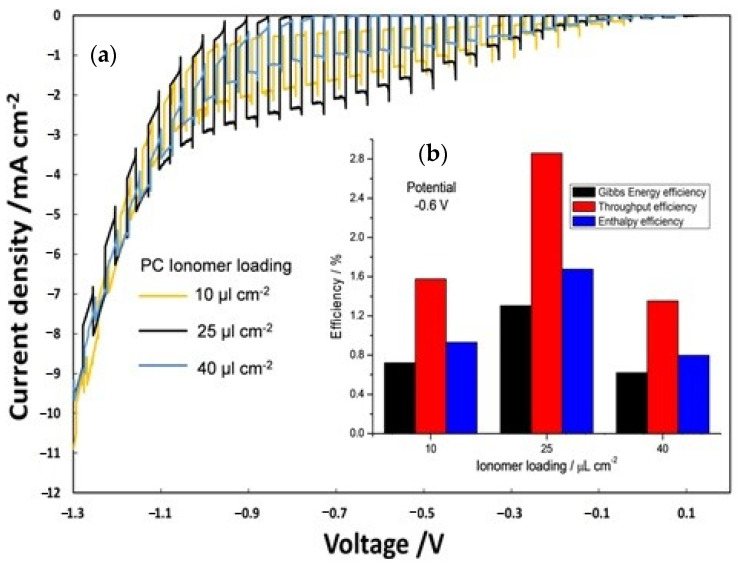
(**a**) On–off polarization curves by varying ionomer loading at photocathode; (**b**) efficiency versus photocathode ionomer content.

A similar trend was shown for the on–off polarization (Figure 7a) and consequently for the efficiency (Figure 7b) of PECs in which the influence of different ionomer loading on the photocathode (10, 25 and 40 µL cm^−2^) was evaluated keeping constant at 25 µL cm^−2^ the photoanode ionomer content.

Also for the photocathode, the best ionomer loading was 25 µL cm^−2^. In this case, the best performing cell showed enthalpy, throughput, and Gibbs energy efficiencies which are about 45% and 53% larger than the cells based on 10 and 40 µL cm^−2^ ionomer dispersions, respectively.

According to these results, an optimized ionomer dispersion obtained by using n-propanol/ethanol (1:1 wt.) with a 25 µL cm^−2^ ionomer loading for both the photoanode and the photocathode was found to be the optimal formulation to maximize the performance of the PEC based on anion-exchange membrane.

A good durability of anionic membrane-based-tandem cell was demonstrated in our previous work [26,27] by means of chrono-amperometric tests at −0.6 V and −0.8 V for 10 h in a proper hydrated environment. Moreover, in these recent activities, a step change in efficiency was achieved passing from FTO to GDL cathode substrate.

Thus, scalability of the overall tandem cell in which earth-abundant semiconductors are deposited onto FTO support, at the photoanode, and hydrophobized GDL substrate, at the photocathode, should be easily realized to obtain efficient and durable production of dry hydrogen from PEC WS process.

## 4. Conclusions

Anion-exchange membrane and ionomer obtained by dissolving the solid shredded membrane in appropriate alcoholic solvents were investigated and optimized for PEC WS applications in a low-cost tandem cell. The anionic membrane was based on polysulfone polymer, containing positive fixed functionalities on the side chains of the polymeric network, particularly quaternary ammonium species counterbalanced by hydroxide ions. FAA3-50 in OH form has shown an IEC value of 1.59 meq/g against a nominal value of 1.85 meq/g, 25 mS/cm of anion conductivity and good transparency at wavelength above 600 nm. Furthermore, hydrogen crossover through the membrane was less than 0.1%. To optimize the ionomer in terms of both dispersion and loading, a low-cost tandem PEC cell, formed by titanium-doped hematite and ionomer at the photoanode and cupric oxide and ionomer at the photocathode, separated by FAA3-50 in OH form, was developed. By using n-propanol/ethanol (1:1 wt.) as solvent for ionomer dispersion and with a 25 µL cm^−2^ ionomer loading at both photoanode and photocathode, enthalpy efficiency of 1.7%, throughput efficiency of 2.9%, and Gibbs energy efficiency of 1.3% were obtained.

## Figures and Tables

**Table 1:** Active groups of FAA3-50 membrane with different counter-ions; Br^−^, Cl^−^, OH^−^.

*Membrane Counter-Ion*	*IEC, meq/g*	*Active Groups, %*
Br^−^	2.28	120
Cl^−^	1.85	100
OH^−^	1.59	86

**Table 2:** Electro-chemical H_2_ crossover of FAA3-50-based MEA.

*T, °C*	*H_2_ Crossover, mA/cm^2^*	*H_2_ Crossover, mol/s cm^2^*	*H_2_ Crossover, ml/s cm^2^*	*H_2_ Crossover, %*
40	0.64	3.32E−09	7.43E−05	0.037
50	0.56	2.90E−09	6.50E−05	0.035
60	0.68	3.52E−09	7.90E−05	0.046

## Supplementary Materials

The following are available online at https://www.mdpi.com/2073-4360/12/12/2991/s1, Figure S1: LSV plots at the 3 different temperatures.
