# Supplementary material for: Anionic Exchange Membrane for Photo-Electrolysis Application
Source: Polymers (Basel). 2020 Dec 15;12(12):2991. doi: 10.3390/polym12122991 (PMC7765393; doi:10.3390/polym12122991)
Supplement: Supplementary file 1 [file polymers-12-02991-s001.pdf]

polymers

MDPI

Supplementary Information

Anionic Exchange Membrane for Photo-Electrolysis Application

Carmelo Lo Vecchio *, Alessandra Carbone *, Stefano Trocino, Irene Gatto, Assunta Patti, Vincenzo Baglio, and Antonino Salvatore Aricò.

Institute for Advanced Energy Technologies "Nicola Giordano" - CNR-ITAE, Via Salita S. Lucia sopra Contesse 5 - 98126 Messina, Italy; carmelo.lovecchio@itae.cnr.it (C.L.V.); alessandra.carbone@itae.cnr.it (A.C.); stefano.trocino@itae.cnr.it (S.T.); irene.gatto@itae.cnr.it (I.G.); assunta.patti@itae.cnr.it (A.P.); vincenzo.baglio@itae.cnr.it (V.B.); antonino.arico@itae.cnr.it (A.S.A.)

* Correspondences: carmelo.lovecchio@itae.cnr.it (C.L.V.); alessandra.carbone@itae.cnr.it (A.C.); Tel.: +39-090-624-288 (C.L.V.); +39-090-624-273 (A.C.)

Received: 19 November 2020; Accepted: 12 December 2020; Published: 15 December 2020

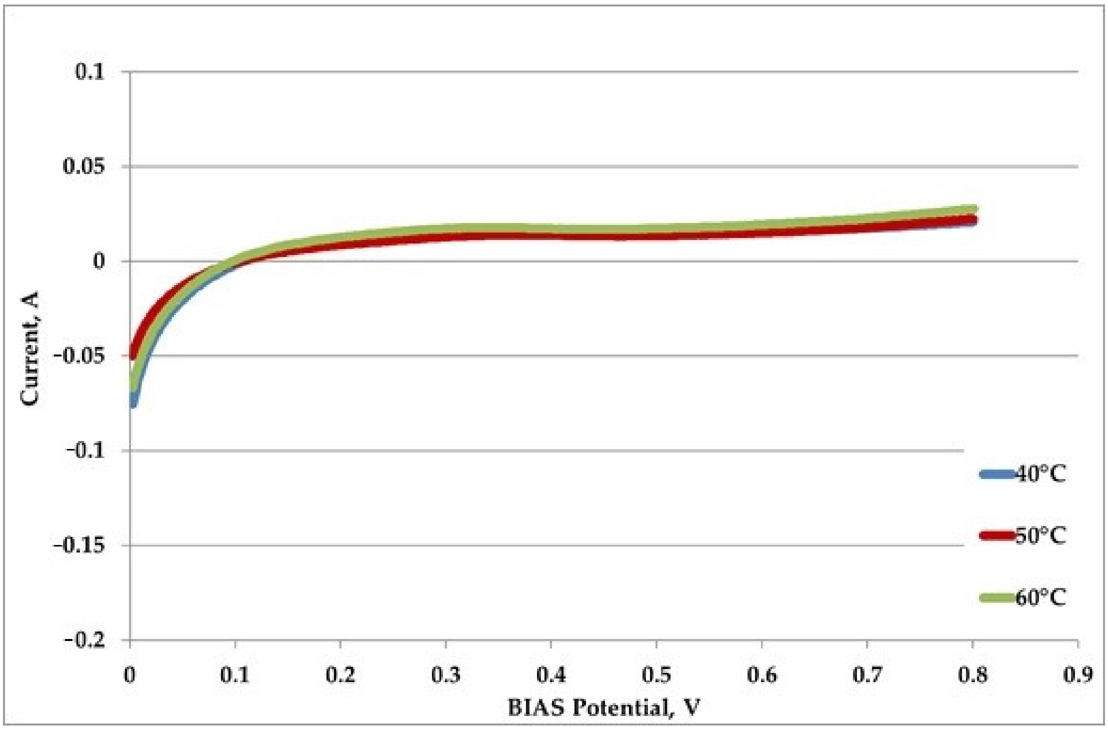

Figure S1. LSV plots at the 3 different temperatures.

© 2020 by the authors. Licensee MDPI, Basel, Switzerland. This article is an open access article distributed under the terms and conditions of the Creative Commons Attribution (CC BY) license (http://creativecommons.org/licenses/by/4.0/).
